# Herpes zoster incidence in Germany - an indirect validation study for self-reported disease data from pretest studies of the population-based German National Cohort

**DOI:** 10.1186/s12879-019-3691-2

**Published:** 2019-01-30

**Authors:** Mahrrouz Caputo, Johannes Horn, André Karch, Manas K. Akmatov, Heiko Becher, Bettina Braun, Hermann Brenner, Stefanie Castell, Beate Fischer, Guido Giani, Kathrin Günther, Barbara Hoffmann, Karl-Heinz Jöckel, Thomas Keil, Birgit Klüppelholz, Lilian Krist, Michael F. Leitzmann, Wolfgang Lieb, Jakob Linseisen, Christa Meisinger, Susanne Moebus, Nadia Obi, Tobias Pischon, Sabine Schipf, Börge Schmidt, Claudia Sievers, Astrid Steinbrecher, Henry Völzke, Rafael Mikolajczyk

**Affiliations:** 1 Department of Epidemiology, Helmholtz Centre for Infection Research, Inhoffenstraße 7, 38124 Braunschweig, Germany; 2 Institute for Medical Epidemiology, Biometry, and Informatics (IMEBI), Medical Faculty of the Martin Luther University Halle-Wittenberg, Magdeburger Str. 8, 06110 Halle (Saale), Germany; 3 German Centre for Infection Research (DZIF), Hannover-Braunschweig site, Braunschweig, Germany; 4 Institute for Epidemiology and Social Medicine, University of Münster, Domagkstraße 3, 48149 Münster, Germany; 5 TWINCORE, Centre for Experimental and Clinical Infection Research, Hannover, Germany; 6 Institute of Public Health, University Hospital Heidelberg, Im Neuenheimer Feld 324, 69120 Heidelberg, Germany; 7 Institute for Medical Biometry and Epidemiology, University Medical Center Hamburg-Eppendorf, Martinistraße 52, 20246 Hamburg, Germany; 8 Division of Clinical Epidemiology and Aging Research, German Cancer Research Center (DKFZ), Im Neuenheimer Feld 581, 69120 Heidelberg, Germany; 9 Network Aging Research University of Heidelberg, Bergheimer Straße 20, 69115 Heidelberg, Germany; 10 Department of Epidemiology and Preventive Medicine, University Regensburg, Franz-Josef-Strauß-Allee 11, 93053 Regensburg, Germany; 11 German Diabetes Center (DDZ), Leibniz Research for Diabetes, Auf’m Hennekamp 65, 40225 Düsseldorf, Germany; 12 Leibniz Institute for Prevention Research and Epidemiology – BIPS, Achterstraße 30, 28359 Bremen, Germany; 13 Institute for Occupational, Social and Environmental Medicine, Heinrich-Heine-University of Düsseldorf, POB 101007, 40001 Düsseldorf, Germany; 14 Institute of Medical Informatics, Biometry and Epidemiology, University Hospital Essen, Hufelandstraße 55, 45147 Essen, Germany; 15 Institute for Social Medicine, Epidemiology and Health Economics, Charité-Universitätsmedizin Berlin, Luisenstraße 57, 10117 Berlin, Germany; 16 Institute of Epidemiology, Christian-Albrechts-University Kiel, Niemannsweg 11, 24105 Kiel, Germany; 17 Helmholtz Zentrum Munich, German Research Center for Environmental Health, Institute of Epidemiology II, Ingolstädter Landstraße 1, 85764 Neuherberg, Germany; 18 Chair of Epidemiology, LMU Munich, UNIKA-T, Neusässer Straße 47, 86156 Augsburg, Germany; 19 Centre of Urban Epidemiology, Institute of Medical Informatics, Biometry and Epidemiology, University Hospital Essen, Hufelandstraße 55, 45147 Essen, Germany; 20 Department of Cancer Epidemiology/Clinical Cancer Registry and Institute for Medical Biometrics and Epidemiology, University Clinic Hamburg-Eppendorf, Martinistraße 52, 20246 Hamburg, Germany; 21 Molecular Epidemiology Research Group, Max-Delbrück-Center for Molecular Medicine in the Helmholtz Association (MDC), Robert-Rössle-Straße 10, 13125 Berlin-Buch, Germany; 22 Institute for Community Medicine, University Medicine Greifswald, University Medicine Greifswald, Walther-Rathenau-Str. 48, 17475 Greifswald, Germany

**Keywords:** German National Cohort, Incidence, Self-reports, Validity, Herpes zoster, Postherpetic neuralgia

## Abstract

**Background:**

Until now, herpes zoster (HZ)-related disease burden in Germany has been estimated based on health insurance data and clinical findings. However, the validity of self-reported HZ is unclear. This study investigated the validity of self-reported herpes zoster (HZ) and its complication postherpetic neuralgia (PHN) using data from the pretest studies of the German National Cohort (GNC) in comparison with estimates based on health insurance data.

**Methods:**

Data of 4751 participants aged between 20 and 69 years from two pretest studies of the GNC carried out in 2011 and 2012 were used. Based on self-reports of physician-diagnosed HZ and PHN, age- and sex-specific HZ incidence rates and PHN proportions were estimated. For comparison, estimates based on statutory health insurance data from the German population were considered.

**Results:**

Eleven percent (95%-CI, 10.4 to 12.3, *n* = 539) of the participants reported at least one HZ episode in their lifetime. Our estimated age-specific HZ incidence rates were lower than previous estimates based on statutory health insurance data. The PHN proportion in participants older than 50 years was 5.9% (1.9 to 13.9%), which was in line with estimates based on health insurance data.

**Conclusion:**

As age- and sex-specific patterns were comparable with that in health insurance data, self-reported diagnosis of HZ seems to be a valid instrument for overall disease trends. Possible reasons for observed differences in incidence rates are recall bias in self-reported data or overestimation in health insurance data.

**Electronic supplementary material:**

The online version of this article (10.1186/s12879-019-3691-2) contains supplementary material, which is available to authorized users.

## Background

Herpes zoster (HZ, also known as shingles) is a painful skin rash with blisters in a localized area, which is caused by the reactivation of a latent varicella zoster virus (VZV) infection [1]. Since HZ mostly affects elderly individuals, the number of HZ cases will increase in the next decades due to demographic changes in developed countries [2], which are characterized by decreasing fertility rates and increasing life expectancy leading to considerable changes in the age structure of societies. About 5 to 30% of subjects with HZ experience postherpetic neuralgia (PHN) [3]; the latter is often accompanied by a substantial impairment of quality of life and is associated with considerable health care costs [2]. In large-scale prospective cohort studies (such as the German National Cohort (GNC)), history of HZ is assessed based on face-to-face interviews or patient-administered questionnaires. The validity of self-reported HZ diagnoses obtained in this way is, however, unclear. Previous studies demonstrated that diseases causing severe long-term restriction of quality of life, which is associated with intensive medical therapies and frequent visits to the physician, are remembered well. This applies to chronic or long-term diseases such as cancer, diabetes, and rheumatoid arthritis, which have been shown to be reported with reasonable accuracy [4–8]. Similar results were also shown for event-type diseases with a strong emotional component and long-term consequences such as stroke or myocardial infarction [8–13]. In contrast, HZ without PHN has no long-term consequences, and even the treatment of a PHN episode is predominantly temporarily limited [14]. Based on these considerations, findings on validity of reporting for other diseases might not be applicable to HZ, making a separate assessment necessary. Typically, the validity of self-reported diagnoses is assessed by directly comparing diagnoses on individual level with a gold standard (such as, medical records [7, 15], inquiry of the primary physician [16, 17], or a physical examination [4, 18]). An alternative method of indirect validation is the comparison of aggregated disease frequency measures with other studies or data sources [19], particularly if they are collected at population level. We have realized this approach by using the comprehensive data set of the pretest studies of the GNC. The aim was to assess the validity of self-reported diagnoses of HZ by comparing our estimates at population level with those derived from studies based on statutory health insurance data in Germany.

## Methods

### Data source

The GNC is a nationwide prospective population-based cohort study with an anticipated number of 200,000 participants recruited in 18 study centers in Germany; the baseline assessment started in 2014 [20]. For planning and preparing of the GNC, two cross-sectional feasibility studies (pretests 1 and 2) were carried out in 2011 and 2012, respectively. The participants were recruited via age-stratified random sampling from regional population registration offices. The recruitment strategy characteristics varied between study centers, but procedures were similar across study centers [21]. The response proportions ranged from 10 to 51% depending on study center [21, 22]. In order to obtain the necessary number of study participants in certain age strata, additionally a small proportion of convenience participants (less than 10%) were enrolled in some study centers. Study participants performed computer-assisted face-to-face interviews to assess their medical history, socio-demographic and economic characteristics, and they underwent various medical examinations; moreover, biological samples such as blood, urine, stool, nasal and pharyngeal swabs were collected.

In the current analysis 2647 participants of pretest 1 and 2897 participants of pretest 2 were included. For the assessment of HZ disease status, the following questions from the core interview were used:

Question 1: *“Have you ever been diagnosed with shingles (herpes zoster) by a physician?”*

Question 2: *“Have you been diagnosed with shingles (herpes zoster) by a physician in the last twelve months?”*

And if question 1 was answered with yes:

Question 3: *“At what age (in which year) have you been diagnosed with shingles (herpes zoster)?”*

Question 4: *“Have you ever been diagnosed by a physician with postherpetic neuralgia as a complication of shingles (herpes zoster)?”*

PHN (as assessed by question 4) was considered only in pretest 2 and was defined as “*…severe pain in the area of shingles-rash, lasting longer than 4 months*”. Only participants answering question 1 with *“Yes”* could specify if they had ever been diagnosed with PHN. The answer options to questions 1, 2, and 4 were “*Yes*”, “*No*”, and “*Don’t know*”. Regarding question 3, the participants could either report the year of diagnosis or the age at the time of diagnosis of HZ.

Pretest study participants were included in this analysis if information on age, sex, and at least one question regarding their HZ disease history was available. Participants outside the intended age-range for the GNC (20 to 69 years) were excluded from the current analysis.

Estimates based on health insurance data from three different studies were used for comparison, described in detail in Table 1 [2, 23, 24].

**Table 1:** Studies using statutory health insurance data of the German population for estimating HZ incidence rates

First author (year)	Data collection year	Data source	HZ incidence rate per 1000 PY (95%-CI)
Ultsch (2013) [2]	2004 to 2009	SHI AOK Hesse/KV Hesse	5.8 (5.6 to 5.9)
Ultsch (2011) [23]	2007 to 2008	ASHIP database^a^	9.6 (9.6 to 9.6)^b^
Hillebrand (2014) [24]	2005 to 2009	GePaRD^c^	6.0 (5.9 to 6.0)

*AOK* “Allgemeine Ortskrankenkasse”, *ASHIP* Association of Statutory Health Insurance Physicians, *GePaRD* German Pharmacoepidemiological Research Database, *CI* confidence interval, *HZ* herpes zoster, *KV* Regional Association of SHI-Accredited Physicians, *SHI* statutory health insurance

^a^ASHIP organized in 17 units largely consistent with 16 federal states of Germany; in 2007 data on 14 ASHPIs and in 2008 data on 11 ASHIPs

^b^Only subjects over 50 years of age included

^c^Data from three SHIs

### Statistical analysis

After excluding individuals with missing data on history of HZ, age or sex we performed a complete case analysis. Firstly, using the pretest data, we directly estimated the crude annual HZ incidence rate (IR) based on the presence of HZ in the past 12 months (question 2). Secondly, using reported diagnosed HZ cases (question 1) and the age at diagnosis (question 3), we calculated the cumulative incidence of HZ, taking into account all HZ cases reported up to a given age, divided by the number of individuals of that age or older. In case of missing information regarding age at HZ diagnosis, we performed an imputation. We used proportions according to the distribution of age at HZ diagnosis among individuals up to 5 years older/younger than the individual with missing information as weights. We further assessed whether there was a difference in the reported cumulative incidence of HZ for four different birth cohorts. For this purpose, we subdivided the study population into cohorts born in 10-year intervals. To investigate whether sex differently affects the hazard of HZ in the 10-year cohort, we used Cox regression. Thirdly, based on the data collected in questions 1 and 3, we estimated IR of HZ in 10-year age-groups by dividing cases occurring in the respective age-bands by the corresponding person-time (censored at age of HZ or age at interview for those not reporting HZ). Subsequently, we compared IR calculated by this approach with IR from three studies using health insurance data from the German population [2, 23, 24]. Fourthly, we assessed the proportion of participants with HZ who experienced PHN and compared it with estimates based on health insurance data [2]. Study participants answering “*Don’t know*” on the question of whether they had HZ were excluded from the main analysis. The statistical analyses were performed using SAS version 9.3 (Basic, SAS Institute Inc., Cary, North Carolina, USA) and R (version 3.2.4).

## Results

### Baseline characteristics of the study population

**Fig. 1:**
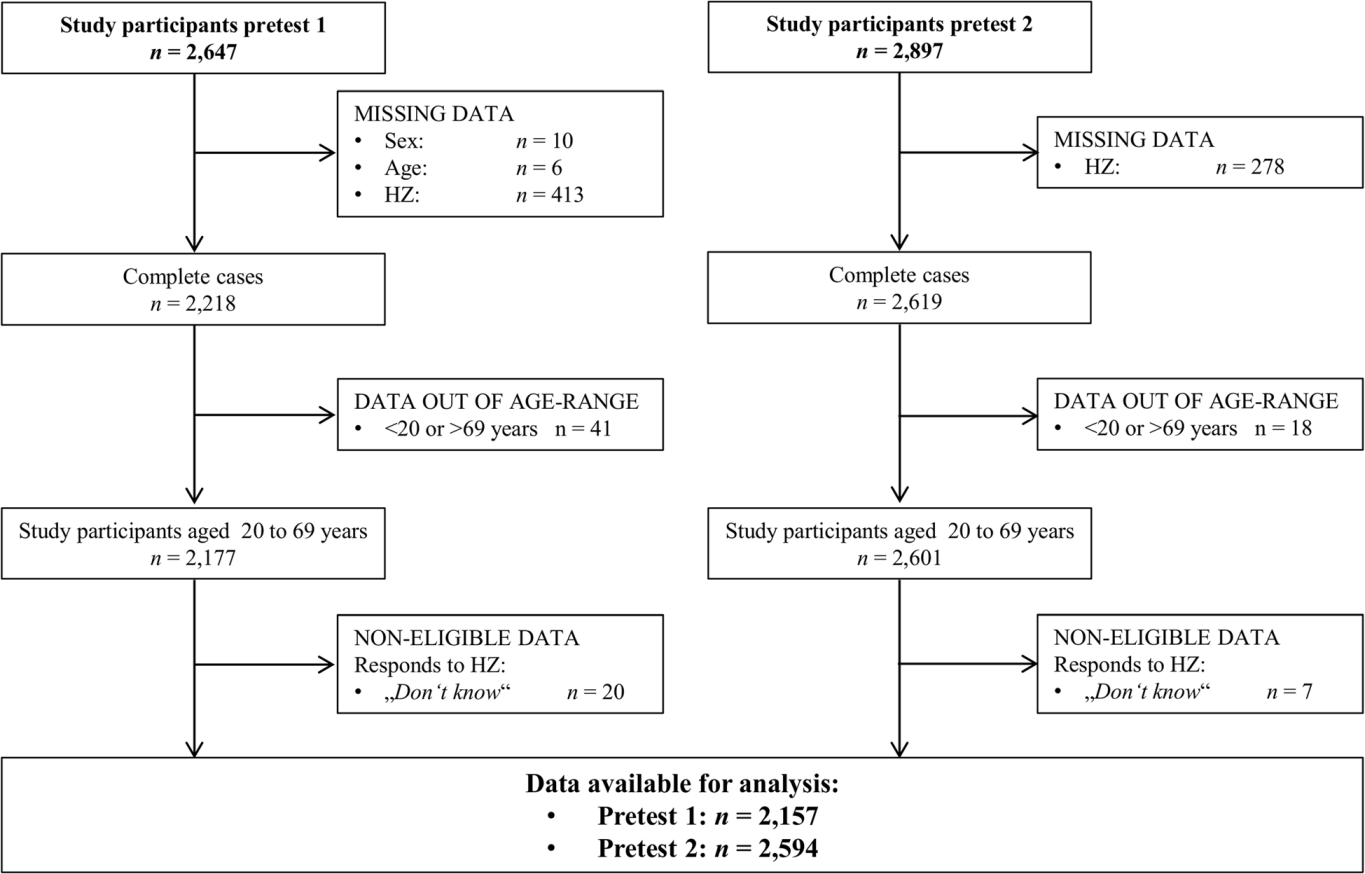
Flow chart of the study population

After assessment of inclusion and exclusion criteria, 4751 (85.7%) of the 5544 pretest participants were included in the analysis (Fig. 1). The most frequent reason for exclusion was missing information on HZ status (*n* = 691), since some study centers did not implement the questions on HZ disease history for parts of or even for the entire pretest study period. In addition, 59 participants did not meet the predefined GNC age-range of 20 to 69 years. Also, 27 participants who stated that they did not know whether they had ever experienced HZ were excluded from the current analysis. The proportion of female participants was slightly higher in both pretest studies (54.9%) than in the German population of this age-range (50.0%) (Table 2). Most of the participants were in the age-group 60 to 69 years (32.7% of all participants). The majority of participants had a higher education entrance qualification (46.5%). Eleven percent (*n* = 539) of participants reported having ever been diagnosed with HZ.

**Table 2:** Baseline characteristics of included study participants of pretest 1 and 2 of the GNC

Variables	Male (*n* = 2145) *n* (%)	Female (*n* = 2606) *n* (%)	Total (*n* = 4751) *n* (%)
Age-group in years			
20 to 29	170 (7.9)	233 (8.9)	403 (8.5)
30 to 39	212 (9.9)	249 (9.6)	461 (9.7)
40 to 49	481 (22.4)	601 (23.1)	1082 (22.8)
50 to 59	542 (25.3)	709 (27.2)	1251 (26.3)
60 to 69	740 (34.5)	814 (31.2)	1554 (32.7)
Median age in years (IQR)	53 (44 to 62)	52 (43 to 61)	53 (43 to 62)
Education level^a^			
High	1067 (49.7)	1143 (43.9)	2210 (46.5)
Intermediate	300 (14.0)	596 (22.9)	896 (18.9)
Low	717 (33.4)	785 (30.1)	1502 (31.6)
Without school-leaving certificate	29 (1.4)	35 (1.3)	64 (1.3)
Missing data	32 (1.5)	47 (1.8)	79 (1.7)
Herpes zoster cases	209 (9.7)	330 (12.7)	539 (11.3)

*IQR* interquartile range

^a^Low: without school-leaving certificate or lower secondary school certificate; intermediate: secondary school certificate; high: general qualification for university entrance

### Crude annual herpes zoster incidence rate

Twenty-nine participants reported a HZ episode in the past 12 months, resulting in a crude IR of 6.2 per 1000 PY (95%-CI: 3.9 to 8.4) for both pretest studies combined.

### Cumulative incidence of herpes zoster

**Fig. 2:**
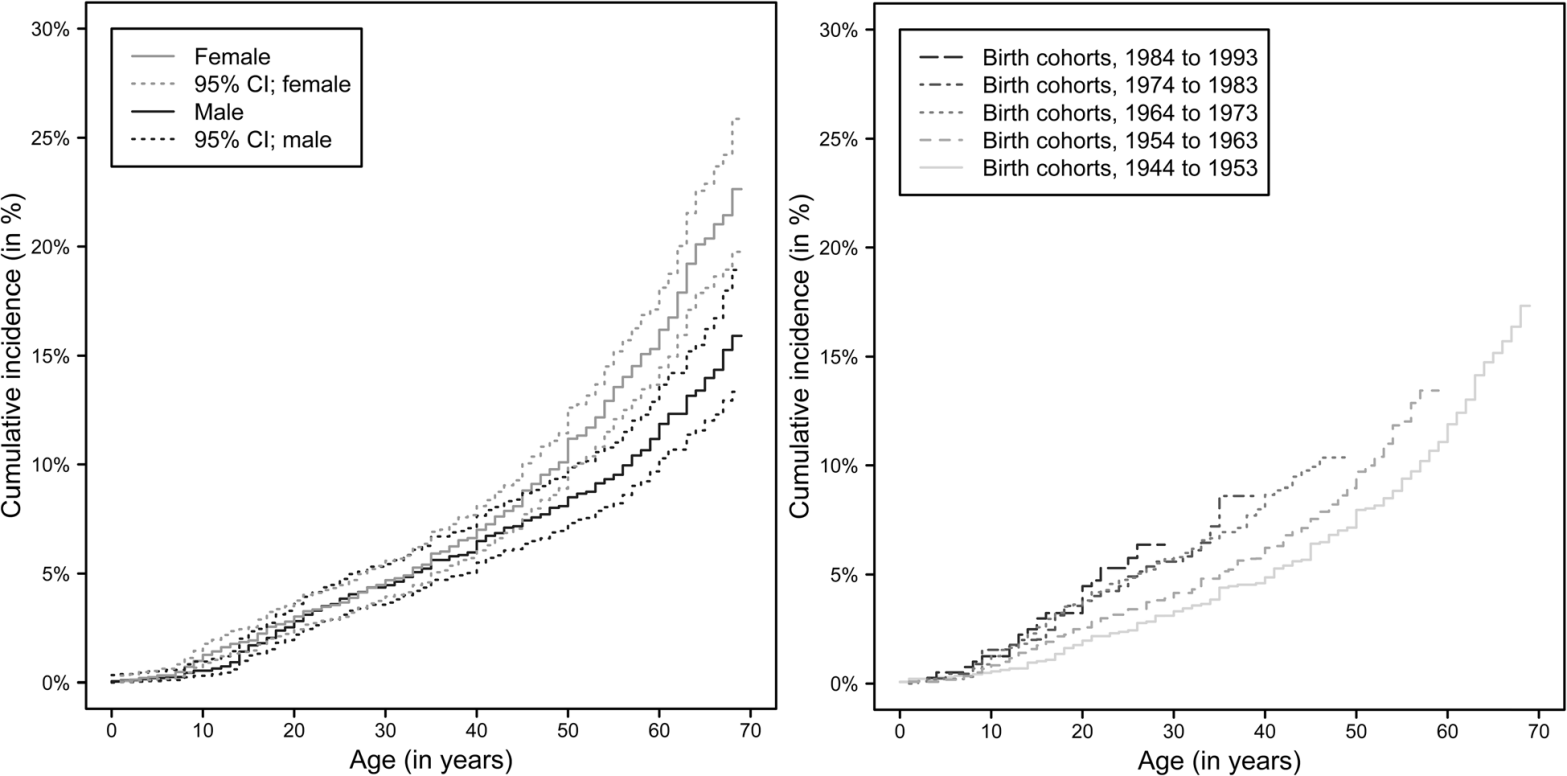
Cumulative incidence of HZ up to a given age by sex (left) and by birth-cohort (right)

The cumulative incidence of HZ was similar in males and females up to the age of 40 years (Fig. 2, left panel). Above the age of 40 years, the cumulative incidence increase with age was more pronounced among female than male participants, resulting in a cumulative incidence of 22.6% (95%-CI: 19.8 to 25.9%) in females and 15.9% (95%-CI: 13.3 to 18.9%) in males at 69 years of age. This observation was confirmed by the results of the Cox regression analysis (Table 3); up to the age of 40 years, no sex-specific effects on HZ incidence were observed. Above the age of 40 years, female participants had about two times higher hazard of HZ than male participants.

**Table 3:** Effect of sex on HZ incidence stratified by age-group

Age-group (in years)	Hazard Ratio (95%-CI) for female vs male sex^a^	*p*-value
20 to 29	1.05 (0.80 to 1.38)	0.727
30 to 39	1.13 (0.74 to 1.72)	0.569
40 to 49	2.05 (1.36 to 3.13)	< 0.001
50 to 59	1.62 (1.04 to 2.51)	0.031
60 to 69	1.96 (1.05 to 3.66)	0.035

^a^Adjusted for calendar time

The cumulative incidence was higher in the younger birth cohorts (1984 to 1993, 1974 to 1983 and 1964 to 1973) compared to the older birth cohorts (1954 to 1963 and 1944 to 1953) (Fig. 2, right panel).

### Comparison of herpes zoster incidence rates based on self-reports with estimates based on health insurance data

**Fig. 3:**
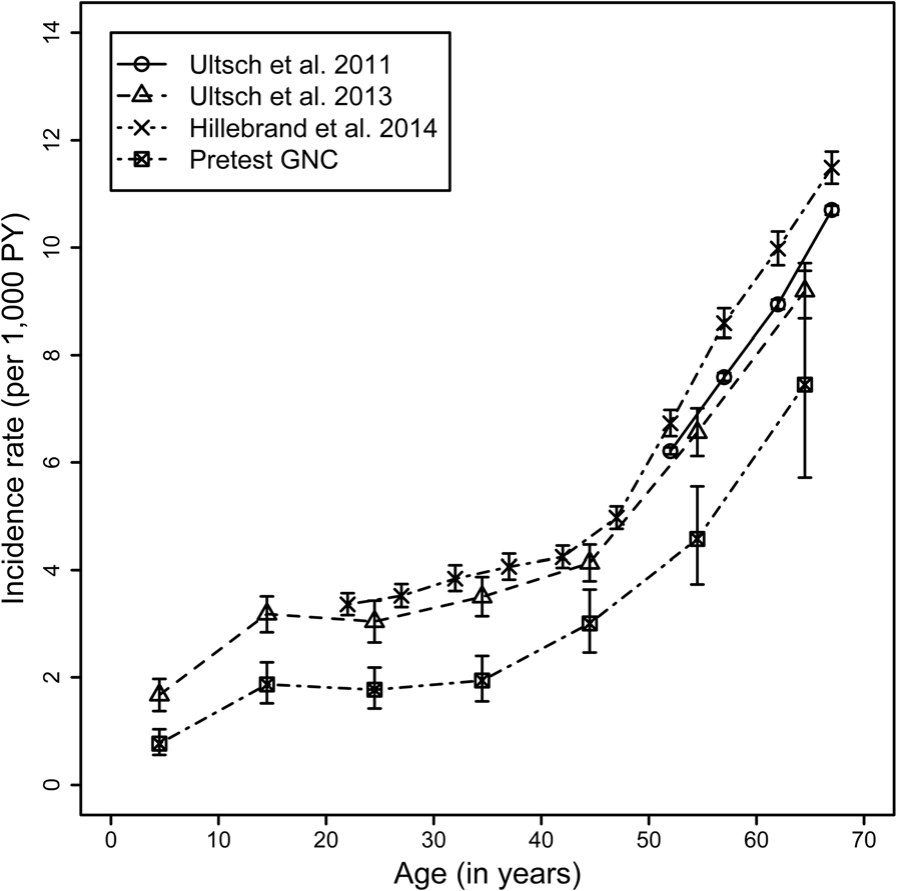
Comparison of incidence rates of HZ from GNC pretest studies with German health insurance data. GNC: German National Cohort; PY: Person-years; Depending on the study, 5- or 10-year age-groups were used. The whiskers indicate 95%-CI

Previous studies using health insurance data in Germany (Table 1) reported all similar age-specific IR for HZ [2, 23, 24] (Fig. 3). In comparison to those estimates, the age-specific IR estimates based on self-reports in the pretest studies of the GNC were considerably lower. However, the age-dependent increase of IR was similar to that observed in health insurance data; the difference between self-reported data and health insurance data was approximately constant across age-groups. This also applies to sex-specific differences with higher rates in females than males, especially above the age of 40 years (Additional file 1). A sensitivity analysis was performed by reclassifying participants who answered “*Don’t know*” into the “*No*” group, which produced slightly smaller cumulative incidences and IR (data not shown).

### Proportion of herpes zoster cases developing postherpetic neuralgia

Information about PHN was collected only in pretest 2 of the GNC. Of the 291 participants with a HZ episode, 10 reported to have suffered from PHN (3.4%; 95%-CI: 1.7 to 6.3%); this was less common than in studies using health insurance data [24–26]. The proportion of PHN in participants aged over 50 years was 5.9% (95%-CI: 1.9 to 13.9%), which is in line with one of the studies used for comparison [2].

## Discussion

We investigated the validity of self-reported diagnoses of HZ in the pretest studies of the GNC by with by comparison with available estimates based on health insurance data. While the age-specific pattern of the IR was correctly reflected in the self-reported data, the estimated IR in our study were lower compared to IR obtained from health insurance data. Moreover, we found evidence for a birth cohort effect with higher HZ incidence in younger individuals.

### Comparison with previous studies

A direct estimation of age- and sex-specific IR of HZ based on the reported incidence in the past 12 months was precluded by insufficient sample size. However, given the anticipated large sample size of 200,000 individuals in the GNC [20], these estimates will still represent the best available source of evidence for current cumulative age-specific IR of HZ in Germany. In comparison to IR based on health insurance data [2, 23, 24], the estimates from the GNC were considerably lower across all age-groups. This can be either caused by a selection of healthier participants in the GNC or by recall bias, particularly for episodes of HZ dating back decades. It is also possible that individuals who recently suffered from HZ did not participate in the pretest studies due to poor health conditions as the incidence of HZ is much higher among individuals who suffered from severe immune deficiency disorders (such as HIV or cancer). Selection mechanisms play a small role in health insurance data especially if the study population is not restricted to one very specific health insurance company. The assumption of recall bias as an explanation for the observed differences is supported by the observed birth cohort effect. This is in line with previous findings showing that the quality of self-reported diagnoses is affected by the length of recall required (time since most recent health exam) [4, 18] (in addition to factors like sex, age, education, comorbidity, and natural course of the diseases [8, 18, 27, 28]). However, the observed increase in diagnosed IR of HZ could also be a true increase, which would explain the birth cohort effects observed in our study [14]. Several other studies, most of them based on secondary data sources (e.g. outpatient, hospital, insurance) with small potential for recall bias showed an increase in IR of HZ over the past 50 years all over the world [29]. The studies used for comparison in our study were based on health insurance data from 2004 to 2009, while HZ episodes in the GNC were reported by participants for the period between 1944 and 2012. One possible explanation for this increase is the so-called boosting-hypothesis; namely the contact with varicella protects those infected with VZV from reactivation of the virus as HZ. Decreasing fertility, aging population, and the introduction of vaccination against varicella in Germany in 2004 all contribute to reducing the exposure to varicella, which in turn leads to an increase in the number of HZ cases in unvaccinated individuals [29]. A further reason for the observed difference could be related to an overestimation of the true IR of HZ in studies using health insurance data. It has been reported previously (even for HZ [23]) that health insurance data tend to overestimate the true IR in some diseases because they are based on claims data.

### Sex- and age-specific pattern of herpes zoster incidence rates

While age-specific IR of HZ were lower than in the studies used for comparison, the characteristic age-dependent increase of IR of HZ with a substantial rise above 40 years of age was reproduced in the self-reported data; the effect was more pronounced among female than male participants. We identified an effect of sex on HZ incidence above the age of 40 years with female participants having about two times higher hazard of HZ compared to male participants. These sex-specific differences have also been shown in previous studies from Germany. The underlying physiologic reasons, however, have not yet been identified [23, 24]. An advanced age is known to be an important risk factor for HZ as a consequence of immune senescence-induced weakening of the immune response, which promotes a reactivation of latent-persistent VZV [1, 30].

### Estimation of postherpetic neuralgia proportion

Since information on PHN was collected only in pretest 2 and only a small fraction of persons with HZ developed PHN, precision of PHN estimation was low, preventing a stratification by age despite the known strong increase of PHN with age. The literature varies considerably regarding estimates of PHN proportions (4.5 to 20.6%) due to differences in study design, population, and definition of pain duration of PHN [2, 24, 26, 31, 32].

### Strength and limitations

The main strength of our study is the relatively large sample size, including persons contributing self-reported data from various regions of Germany. Moreover, the stratified random sampling of participants from local registration offices in the study regions was population-based. Given the very broad scope of the study, participants are unlikely to have selected themselves into the study based on their interest in HZ. However, several limitations of our study need to be mentioned. Medical records or physician examinations of diagnosed HZ cases were not available for study participants of the pretest studies. Accordingly, we could not attempt a direct validation of disease data against medical records, which is the gold standard for assessing the validity of self-reported data on an individual level. Instead, we compared aggregate measures based on self-reported and health insurance data. A prerequisite for such a comparison is the representativeness of both study populations with respect to the source population; given the relatively low response proportion in our study, this might have not been the case. The use of published data for indirect comparisons represents a simple approach, which is however limited to information available in the sources used as reference. For example, definitions of PHN differed in previous publications; this might explain the heterogeneity of data on this outcome in the literature. As information on the first HZ episode could only be reported in the pretest studies, subsequent HZ episodes could not be considered in the analysis. However, for estimating the IR we censored person-time after the first reported HZ episode under the assumption, that the risk of HZ is not affected by past HZ episodes [33].

## Conclusion

We investigated the validity of self-reported diagnoses of HZ from a population-based study in Germany by comparing them with estimates from health insurance data. We found consistently lower IRs of HZ based on self-reported data compared to health insurance data as well as a birth cohort effect. Age- and sex-specific differences in IRs followed the patterns of estimates based on health insurance data in Germany.

## Additional file

Additional file 1: Herpes zoster incidence in Germany: an indirect validation study for self-reported disease data from the pretest studies of the German National Cohort. Comparison of age-specific incidence rates (per 1000 PY) of herpes zoster from pretest studies of the German National Cohort with studies based on health insurance data in Germany by sex. A: Comparison of incidence rates of herpes zoster (per 1000 PY) in female participants. B: Comparison of incidence rates of herpes zoster (per 1000 PY) in male participants. GNC: German National Cohort. PY: Person-years. (TIF 130 kb)

## Abbreviations

CI: Confidence interval
GNC: German National Cohort
HZ: Herpes zoster
IQR: Interquartile range
IR: Incidence rate
PHN: Postherpetic neuralgia
PY: Person-years
VS: Versus
VZV: Varicella zoster virus
Y: Year
