# Supplementary figures and images for: Herpes zoster incidence in Germany - an indirect validation study for self-reported disease data from pretest studies of the population-based German National Cohort
Source: BMC Infect Dis. 2019 Jan 30;19:99. doi: 10.1186/s12879-019-3691-2 (PMC6354372; doi:10.1186/s12879-019-3691-2)

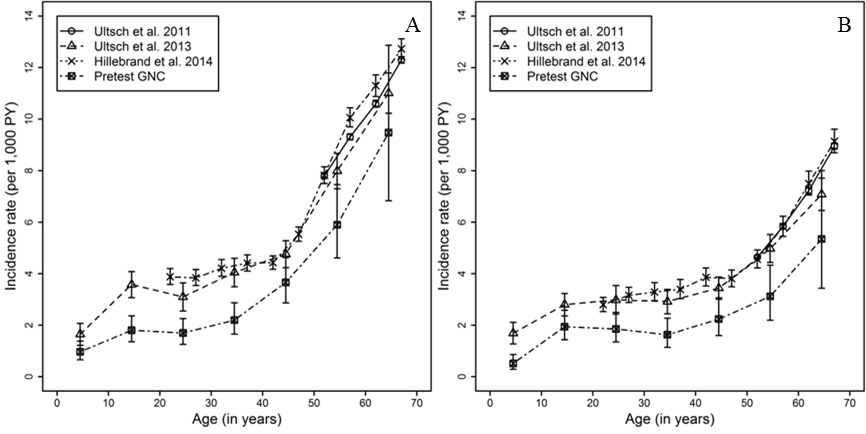

Supplement: Supplementary file 1 — Herpes zoster incidence in Germany: an indirect validation study for self-reported disease data from the pretest studies of the German National Cohort. Comparison of age-specific incidence rates (per 1000 PY) of herpes zoster from pretest studies of the German National Cohort with studies based on health insurance data in Germany by sex. A: Comparison of incidence rates of herpes zoster (per 1000 PY) in female participants. B: Comparison of incidence rates of herpes zoster (per 1000 PY) in male participants. GNC: German National Cohort. PY: Person-years. (TIF 130 kb) [file 12879_2019_3691_MOESM1_ESM.tif]
